# Supplementary material for: Gradual Enhancement of the Assemblage Stability of the Reed Rhizosphere Microbiome with Recovery Time
Source: Microorganisms. 2022 Apr 29;10(5):937. doi: 10.3390/microorganisms10050937 (PMC9146439; doi:10.3390/microorganisms10050937)
Supplement: Supplementary file 1 [file microorganisms-10-00937-s001.zip › microorganisms-1651739-supplementary.pdf]

Supplementary Materials

Supplementary Methods

DNA extraction and PCR amplification

Total DNA was extracted by the CTAB method as follows: A total of 1000 µL of CTAB lysate was added to a 2.0 mL EP tube, lysozyme was added, and then an appropriate amount of sample was added to the lysate. The tube was placed in a 65°C water bath and inverted and mixed several times during the period to fully lyse the sample. The sample was centrifuged, and the supernatant was obtained. A 25:24:1 mixture of phenol (pH 8.0)/chloroform/isoamyl alcohol was added, and the solution was mixed well and centrifuged at 12000 rpm for 10 min. The supernatant was combined with a 24:1 chloroform/isoamyl alcohol solution, mixed well and centrifuged at 12000 rpm for 10 min. The supernatant was added to a 1.5 mL centrifuge tube, isopropanol was added and shaken well, and the mixture was left to settle at -20°C. The solution was centrifuged at 12000 rpm for 10 min, and the supernatant was carefully removed (being careful to not remove the precipitate). The precipitate was washed twice with 1.0 mL of 75% ethanol, and the remaining liquid was collected by centrifugation by pipette. The liquid was blow-dried on a clean bench or air-dried at room temperature (DNA samples were not completely dried). Distilled water was used to dissolve the DNA sample, with incubation at 55-60°C for 10 min. A total of 1 µL of RNase A was added to dissolve the RNA, and the samples were placed at 37°C for 15 min. All PCRs were carried out in 30 µL reactions with 15 µL of Phusion® High-Fidelity PCR Master Mix (New England Biolabs, USA), 0.2 µM forward and reverse primers, and approximately 10 ng of template DNA. Thermal cycling consisted of initial denaturation at 98°C for 1 min, followed by 30 cycles of denaturation at 98°C for 10 s, annealing at 50°C for 30 s, and elongation at 72°C for 30 s. The temperature was held at 72°C for 5 min. The same volume of 1 × loading buffer (containing SYBR green) was mixed with the PCR products, and electrophoresis was performed. Then, mixed PCR products were purified with a Gene JETTM Gel Extraction Kit (Thermo Scientific, Waltham) on 2% agarose gel for detection. PCR products were mixed in equal density ratios and stored at -80°C in a freezer for later use.

Data splitting and filtration

Single-end reads were assigned to samples based on their unique barcode and were truncated by cutting off the barcode and primer sequence. Quality filtering of the raw reads was performed under specific filtering conditions to obtain high-quality clean reads according to the Cutadapt (Martin, 2011) (V1.9.1, http://cutadapt.readthedocs.io/en/stable/) quality control process. The reads were compared with the reference database (Silva database, https://www.arb-silva.de/) (Quast et al., 2013) using the UCHIME algorithm (UCHIME Algorithm, http://www.drive5.com/usearch/manual/uchime_algo.html) (Edgar et al., 2011) to detect chimera sequences, and then the chimera sequences were removed (Haas et al., 2011). The clean reads were finally obtained.

OTU cluster and species annotation

Sequence analysis was performed by UPARSE software (UPARSE v7.0.1001, http://drive5.com/uparse/) (Edgar, 2013). Sequences with ≥ 97% similarity were assigned to the same OTU. Representative sequences for each OTU were screened for further annotation. For each representative sequence, the Silva database (https://www.arb-silva.de/) (Quast et al., 2013) was used based on the Mothur algorithm to annotate taxonomic information. To study the phylogenetic relationship of different OTUs and the difference in the dominant species in different samples (groups), multiple sequence alignment was conducted using MUSCLE software (Version 3.8.31, http://www.drive5.com/muscle/) (Edgar, 2004). OTU abundance information was normalized using a standard sequence number corresponding to the sample with the fewest sequences. Subsequent analyses of alpha diversity and beta diversity were performed based on these normalized data.

Supplementary Data

Table S1. Latitudes and longitudes and basic information of the sample locations

**Sample ID**	**Recovery Time**	**Latitude and Longitude**	**Physicochemical Parameters**					**Collect Time**
			pH	Cond (us·cm^{-1})	TOC (mg·L^{-1})	TN (mg·L^{-1})	TP (mg·L^{-1})	
SH1	40-y	120.682926 E, 31.638804 N	7.34	289.03	8.62	0.72	0.73	11-13 April, July and November, 2020
SH2		120.684299 E, 31.645573 N	6.58	326.33	13.72	0.96	0.52	
SH3		120.690120 E, 31.638832 N	7.27	289.58	6.84	0.55	0.56	
SJ1	30-y	120.798690 E, 31.555673 N	6.60	675.67	39.13	2.64	0.70	
SJ2		120.803035 E, 31.556443 N	6.76	440.26	16.99	1.25	0.54	
SJ3		120.803760 E, 31.551861 N	6.69	553.63	25.64	1.83	0.57	
TH1	20-y	120.354549 E, 31.322206 N	7.57	236.58	7.38	0.69	0.46	
TH2		120.360653 E, 31.319236 N	7.43	231.54	6.88	0.58	0.46	
TH3		120.365975 E, 31.323250 N	7.42	260.29	8.80	0.75	0.48	
TH4		120.361801 E, 31.329070 N	7.33	201.38	8.60	0.72	0.44	
TH5		120.354774 E, 31.327810 N	8.08	215.78	4.34	0.46	0.52	
NH1	10-y	120.619401 E, 31.596576 N	6.78	361.80	15.07	0.98	0.48	
NH2		120.625366 E, 31.590627 N	6.78	180.28	7.93	0.87	0.41	
NH3		120.632683 E, 31.587259 N	6.13	276.81	11.59	0.93	0.39	

Table S2. Microbial assemblage diversity index (mean ± SE, n = 56) for the 40y, 30y, 20y and 10y of restored wetlands. Different lowercase letters present significant differences ($p < 0.05$) among different sampling areas.

Period	Recovery Time	40-y	30-y	20-y	10-y
Level season	bacteria	260.807±61.198ab	269.452±21.065a	244.929±41.710ac	221.277±65.038bc
	fungi	240.062±44.54bc	281.201±31.464a	217.028±51.201c	273.748±47.156ab
Wet season	bacteria	158.297±51.057a	188.088±68.382a	119.729±41.856b	143.328±72.395a
	fungi	268.790±47.228b	351.854±31.995a	188.455±39.527c	254.734±40.390b
Dry season	bacteria	288.847±47.133a	261.167±20.240ab	233.718±52.687b	233.628±42.613b
	fungi	209.857±54.610a	230.457±50.972a	195.982±49.603a	222.270±59.280a

Table S3. The community stability index in the different recovery periods.

	**40y**	**30y**	**20y**	**10y**	**Sig.**
Level season	0.729 (5313)	0.711(5830)	0.718(4636)	0.811(5495)	
Wet season	0.756 (3869)	0.791(5486)	0.749(3097)	0.769(3449)	
Dry season	0.696 (5611)	0.759(5229)	0.763(4349)	0.733(4839)	
Whole year	0.605(6283)	0.616(5939)	0.613(5694)	0.627(6860)	0.016*
Sig.	0.137	0.112	0.112	0.001**	

Note: (the number of OTU), *indicates significant differences at $p < 0.05$; **indicates significant differences at $p < 0.01$.

Table S4. ANOSIM between the groups, the R values between two groups with (**a**) bacterial and (**b**) fungal assemblages.

(a)

	**40y**	**30y**	**20y**	**10y**
40y	1			
30y	0.084**	1		
20y	0.130**	0.109**	1	
10y	0.122**	0.195**	0.108**	1

(b)

	**40y**	**30y**	**20y**	**10y**
40y	1			
30y	0.151**	1		
20y	0.207**	0.256**	1	
10y	0.163**	0.175**	0.279**	1

Note: **indicates significant differences at $p < 0.01$.

Table S5. The statistics of bacterial and fungal indicators

		Level Season			Wet Season			Dry Season			Whole Year		
	**Periods**	**Total**	**Unique species**	**Proportion (%)**	**Total**	**Unique species**	**Proportion (%)**	**Total**	**Unique species**	**Proportion (%)**	**Total**	**Unique species**	**Proportion (%)**
Bacteria	40y	668	338	50.59	277	105	37.90	1147	564	49.17	1839	714	38.83
	30y	906	494	54.52	805	604	75.03	901	378	41.95	2319	1154	49.76
	20y	675	383	56.74	535	467	87.28	388	255	75.44	1557	1058	67.95
	10y	486	352	72.42	226	127	56.19	530	401	75.66	1104	697	63.13
Fungi	40y	59	55	93.22	116	111	95.68	46	45	97.82	233	198	84.97
	30y	86	79	91.86	185	177	95.67	51	50	98.03	448	389	86.83
	20y	31	29	93.54	40	40	100	28	28	100	163	158	96.93
	10y	112	103	91.96	87	80	91.95	59	59	100	324	289	89.19

Table S6. Numbers of bacteria and fungi in the different modules.

**Module**	**M1**	**M2**	**M6**	**M8**	**total**
Total	194	342	90	69	695
Bacteria	104	342	84	63	593
Fungi	90	0	6	9	105
Core taxa	58	286	35	38	1134
Keystone species	0	22	0	0	0
Proportion (%)	29.89	83.63	38.89	55.07	

Table S7. Taxonomy of keystone species in the reed rhizosphere microbiome

Num	OTU_num	Kindom	Phylum	Class	Order	Family	Genus
1	OTU_12	Bacteria	*Proteobacteria*	*Deltaproteobacteria*	*Desulfarculales*	*Desulfarculaceae*	*Desulfatiglans*
2	OTU_19	Archaea	*Euryarchaeota*	*Methanomicrobia*	*Methanosarcinales*	*Methanosaetaceae*	*Methanosaeta*
3	OTU_20	Bacteria	*Nitrospirae*				
4	OTU_62	Bacteria	*Proteobacteria*	*Deltaproteobacteria*	*Syntrophobacterales*	*Syntrophaceae*	
5	OTU_69	Bacteria	*Proteobacteria*	*Deltaproteobacteria*	*Syntrophobacterales*	*Syntrophaceae*	
6	OTU_93	Bacteria	*Proteobacteria*	*Deltaproteobacteria*	*Syntrophobacterales*	*Syntrophobacteraceae*	
7	OTU_115	Bacteria	*Proteobacteria*	*Deltaproteobacteria*	*Syntrophobacterales*	*Syntrophaceae*	
8	OTU_132	Bacteria	*Acidobacteria*	*unidentified_Acidobacteria*	*unidentified_Acidobacteria*	*unidentified_Acidobacteria*	*unidentified_Acidobacteria*
9	OTU_348	Bacteria	*Proteobacteria*	*Deltaproteobacteria*			
10	OTU_383	Bacteria	*Proteobacteria*	*Deltaproteobacteria*			
11	OTU_488	Bacteria	*Chloroflexi*	*Dehalococcoidia*			
12	OTU_499	Bacteria	*Acidobacteria*				
13	OTU_815	Bacteria	*Proteobacteria*	*Deltaproteobacteria*			
14	OTU_927	Bacteria	*Spirochaetes*	*Spirochaetia*	*Spirochaetales*	*Spirochaetaceae*	
15	OTU_1048	Bacteria	*Bacteroidetes*	*Bacteroidia*	*Bacteroidales*		
16	OTU_2983	Bacteria	*Proteobacteria*	*Deltaproteobacteria*	*Desulfobacterales*	*Desulfobacteraceae*	
17	OTU_3793	Bacteria	*Nitrospirae*				
18	OTU_11129	Bacteria	*Latescibacteria*				
19	OTU_13890	Bacteria	*Acidobacteria*				
20	OTU_17435	Bacteria	*Nitrospirae*				
21	OTU_20759	Bacteria	*Acidobacteria*	*Acidobacteriia*	*Solibacterales*		
22	OTU_22938	Bacteria	*Proteobacteria*	*Deltaproteobacteria*	*Desulfarculales*	*Desulfarculaceae*	*Desulfatiglans*

Table S8. The categories of functional profiles

Number	Code	Name	Functional Feature	Functional Groups
1	COG0438, 0463	Glycosyltransferase involved in cell wall biosynthesis	Carbohydrate transport and metabolism	*Proteobacteria, Gammaproteobacteria, Enterobacterales, Pantoea.* etc.
2	COG0451	Nucleoside-diphosphate-sugar epimerase	Cell motility, Signal transduction mechanisms	*Actinobacteria, Rubrobacterales, Rubrobacteraceae, Rubrobacter.* etc.
3	COG0583	DNA-binding transcriptional regulator, LysR family	Cell wall/membrane/envelope biogenesis	*Firmicutes, Clostridia, Clostridiales.* etc.
4	COG0596	Pimeloyl-ACP methyl ester carboxylesterase		*Actinobacteria, Streptomycetales, Streptomycetaceae, Streptomyces.* etc.
5	COG0642	Signal transduction histidine kinase	Cell wall/membrane/envelope biogenesis, Defense mechanisms	*Cyanobacteria, Oscillatoriales, Coleofasciculaceae.* etc.
6	COG0745	DNA-binding response regulator, OmpR family, contains REC and winged-helix (wHTH) domain	Cell wall/membrane/envelope biogenesis	*Proteobacteria, Gammaproteobacteria.* etc.
7	COG1028	NAD(P)-dependent dehydrogenase, short-chain alcohol dehydrogenase family	Inorganic ion transport and metabolism	*Actinobacteria, Corynebacteriales, Mycobacteriaceae.* etc.
8	COG1032	Radical SAM superfamily enzyme YgiQ, UPF0313 family	Lipid transport and metabolism	*Firmicutes, Clostridia.* etc.
9	COG1309	DNA-binding transcriptional regulator, AcrR family	Lipid transport and metabolism, Secondary metabolites biosynthesis, transport and catabolism, General function prediction only	*Actinobacteria, Mycobacteriaceae.* etc.
10	COG1538	Outer membrane protein TolC		*Proteobacteria, Gammaproteobacteria, Vibrionales, Vibrionaceae.* etc.
11	COG1595	DNA-directed RNA polymerase specialized sigma subunit, sigma24 family	Signal transduction mechanisms	*Actinobacteria, Streptosporangiales, Streptosporangiaceae.* etc.
12	COG0515	Serine/threonine protein kinase		*Firmicutes; Bacilli; Lactobacillales; Streptococcaceae; Streptococcus*
13	COG1629	Outer membrane receptor proteins, mostly Fe transport	Signal transduction mechanisms, Transcription	*Bacteroidetes, Flavobacteriales, Flavobacteriaceae, Flavobacterium.* etc.
14	COG2197	DNA-binding response regulator, NarL/FixJ family, contains REC and HTH domains		*Actinobacteria; Pseudonocardiales; Pseudonocardiaceae;Kibdelosporangium.* etc.
15	COG2204	DNA-binding transcriptional response regulator, NtrC family, contains REC, AAA-type ATPase, and a Fis-type DNA-binding domains	Transcription	*Proteobacteria, Alphaproteobacteria, Rhodobacterales, Rhodobacteraceae.* etc.
16	COG2814	Predicted arabinose efflux permease, MFS family		*Firmicutes, Clostridia, Clostridiales, Clostridiales.* etc.
17	COG1506	Dipeptidyl aminopeptidase/acylaminoacyl peptidase	Amino acid transport and metabolism	*Actinobacteria; Rubrobacteria; Rubrobacterales; Rubrobacteraceae; Rubrobacter.* etc.
18	COG0747	ABC-type transport system, periplasmic component		*Spirochaetes; Leptospirales; Leptospiraceae; Leptospira.* etc.
19	COG1132	ABC-type multidrug transport system, ATPase and permease component	Defense mechanisms	*Firmicutes; Bacilli; Lactobacillales; Lactobacillaceae;Paucilactobacillus.* etc.
20	COG0535	Radical SAM superfamily enzyme, MoaA/NifB/PqqE/SkfB family	General function prediction only	*Firmicutes; Clostridia; Thermoanaerobacterales; Thermoanaerobacteraceae; Thermanaeromonas.* etc.
21	COG4636	Endonuclease, Uma2 family (restriction endonuclease fold)		*Cyanobacteria; Nostocales; Nostocaceae; Nostoc.* etc.

22	COG0491	Glyoxylase or a related metal-dependent hydrolase, beta-lactamase superfamily II		*Proteobacteria; Gammaproteobacteria; Enterobacterales;Morganellaceae; Photorhabdus.* etc.
23	COG0845	Multidrug efflux pump subunit AcrA (membrane-fusion protein)		*Spirochaetes, Leptospiraceae, Leptospira.* etc.
24	COG1024	Enoyl-CoA hydratase/carnithine racemase	-	*Actinobacteria; Corynebacteriales; Gordoniaceae;Gordonia.* etc.
25	COG1357	Uncharacterized protein YjbI, contains pentapeptide repeats	Function unknown	*Proteobacteria; Gammaproteobacteria; Candidatus Kentron; unclassified Candidatus Kentron.* etc.
26	PP	Plant pathogen	Pathotroph	*Immersidiscosia eucalypti; Gibellulopsis nigrescens; Gibberella acuminata; Cercospora; Ophiostoma bicolor; Lasiodiplodia iraniensis; Phlyctochytrium lagenaria.*
27	FP	Fungal parasite		*Syncephalis.*
28	PPSS	Plant pathogen soil saprotroph wood saprotroph		*Fusarium;Fusarium oxysporum.*
29	BPLS	Bryophyte parasite leaf saprotroph wood saprotroph	Pathotroph saprotroph	*Pluteus.*
30	AEUS	Animal endosymbiont undefined saprotroph		*Rhodotorula; Rhodotorula mucilaginosa.*
31	FPUS	Fungal parasite undefined saprotroph	Pathotroph-Saprotroph-Symbiotroph	*Bulleribasidium wuzhishanense.*
32	APEP	Animal pathogen endophyte plant pathogen wood saprotroph		*Alternaria.*
33	OMPP	Orchid mycorrhizal plant pathogen wood saprotroph	Saprotroph-Pathotroph-Symbiotroph	*Rhizoctonia.*
34	EPP	Endophyte plant pathogen	Pathotroph symbiotroph	*Colletotrichum; Cladosporium cladosporioides; Colletotrichum coccodes; Colletotrichum destructivum.*
35	APCE	Animal pathogen clavicipitaceous endophyte fungal parasite		*Cordyceps.*
36	DSUS	Dung saprotroph undefined saprotroph wood saprotroph		*Chaetomium.*
37	US	Undefined saprotroph		*Hypocreales; Pseudeurotium; Immersiella caudata; Pseudoproboscispora; Dactylonectria macrodidyma; Platystomum crataegi; Pseudeurotium; Nectriaceae; Exophiala; Mucor; Lopadostoma meridionale; Melanopsamma xylophila; Scolecobasidium humicola; Dactylonectria macrodidyma; Nigrospora; Nectriaceae; Starmera; Diutina mesorugosa; Eurotiales; Hypocreales; Orbilia; Acrocalymma; Idriella lunata; Uwebraunia_dekkeri; Candida.*
38	WS	Wood saprotroph	Saprotroph	*Pseudoproboscispora; Psathyrella typhae; Immersiella caudata; Sphaerellopsis filum; Morchella eximia.*
39	LS	Leaf saprotroph		*Crinipellis rhizomaticola.*
40	DSSS	Dung saprotroph soil saprotroph wood saprotroph		*Ascobolaceae.*
41	USWS	Undefined saprotroph wood saprotroph		*Rhinocladiella.*
42	SS	Soil saprotroph		*Geastrum lageniforme.*
43	DS	Dung saprotroph		*Cercophora coprophila.*
44	EUS	Ectomycorrhizal undefined saprotroph	Saprotroph symbiotroph	*Entoloma.*
45	AM	Arbuscular mycorrhizal	Symbiotroph	*Glomeromycota; Archaeosporaceae; Glomeraceae; Acaulosporaceae; Diversisporaceae; Acaulospora mellea; Paraglomerales; Diversispora.*
46	LC	Lichenized		*Sticta; Trichosporon asahii; Acaulospora mellea.*

47	EM	Ectomycorrhizal		*Astraeus; Ceratobasidium; Pachyphloeus; Mallocybe; Lactarius pomiolens; Otidea daliensis; Hebeloma hiemale; Lactarius pomiolens; Inocybe semifulva; Inocybe lacunarum; Hydnum vesterholtii.*
48	UA	Unassigned	Unassigned	*Ascomycota; GS01; Rozellomycota; Chytridiomycota; Rozellomycota; Sordariales; Pezizaceae; Sordariomycetes; Pleosporales; Thelephoraceae; Helotiales; Lasiosphaeriaceae; Dothideomycetes; Halosphaeriaceae; Dothideomycetes; Sebacinales; Ceratobasidiaceae; Xylariales; Polyporaceae; Phomatodes; Plectosphaerellaceae;Leucosporidium intermedium; Pseudopithomyces maydicus; Vanrija humicola; Phialemoniopsis curvata; Gonapodya polymorpha.*

Note: functional profiles are depicted according to the NCBI database (https://www.ncbi.nlm.nih.gov/).

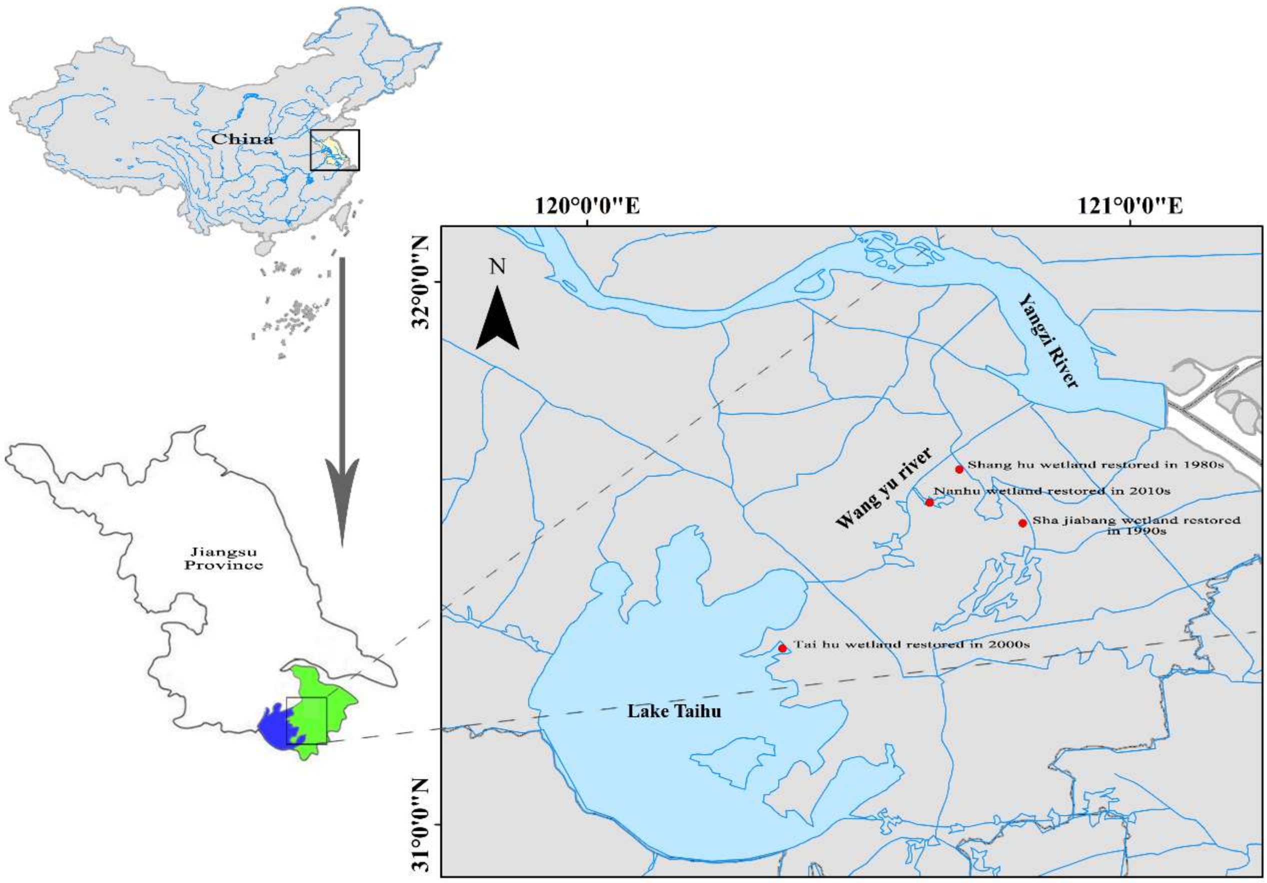

Figure S1. Location of the restored wetlands in the Lake Taihu Basin, East, China.

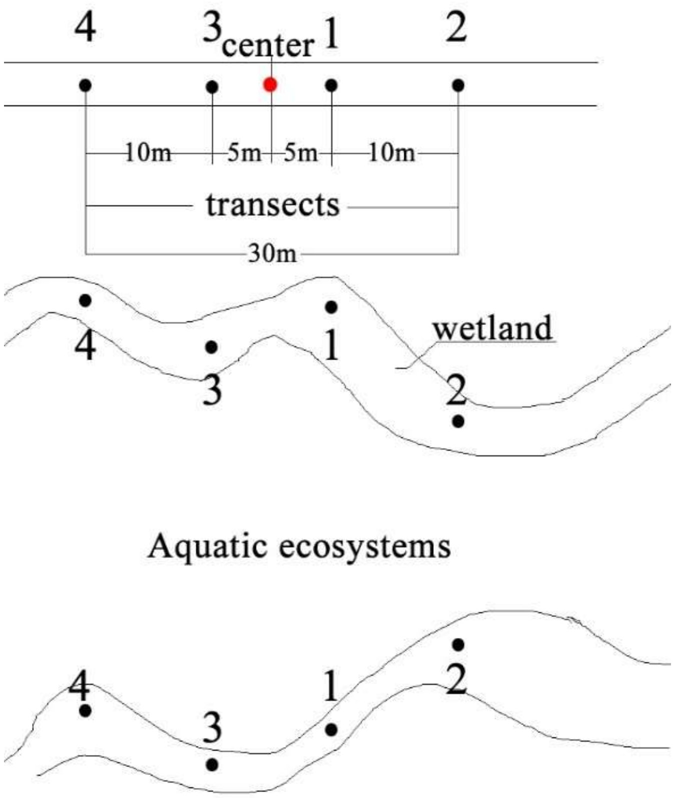

Figure S2. Schematic of the sample locations.

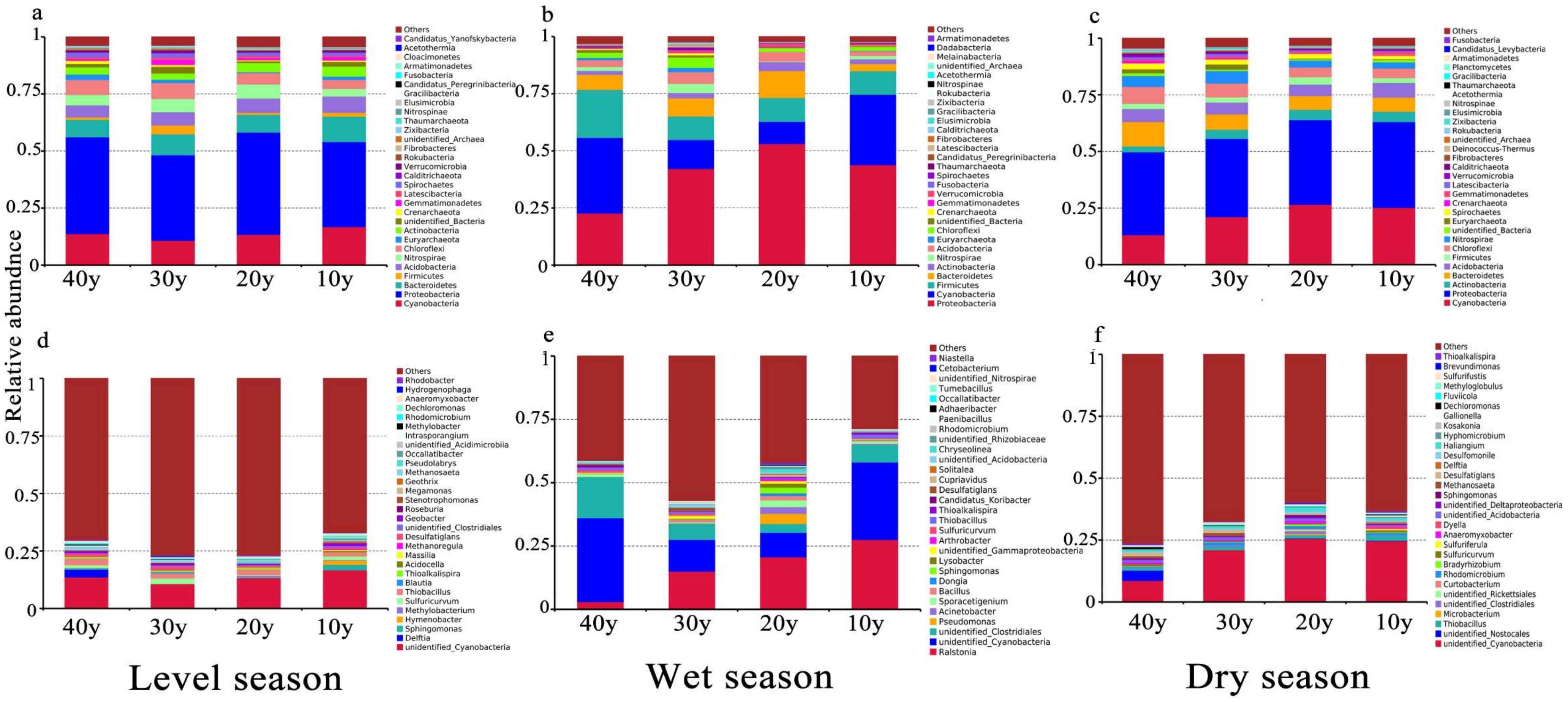

Figure S3. The relative abundance of bacterial communities at the (a-c) phylum and (d-f) genus- levels in the reed rhizosphere during the level, wet and dry seasons.

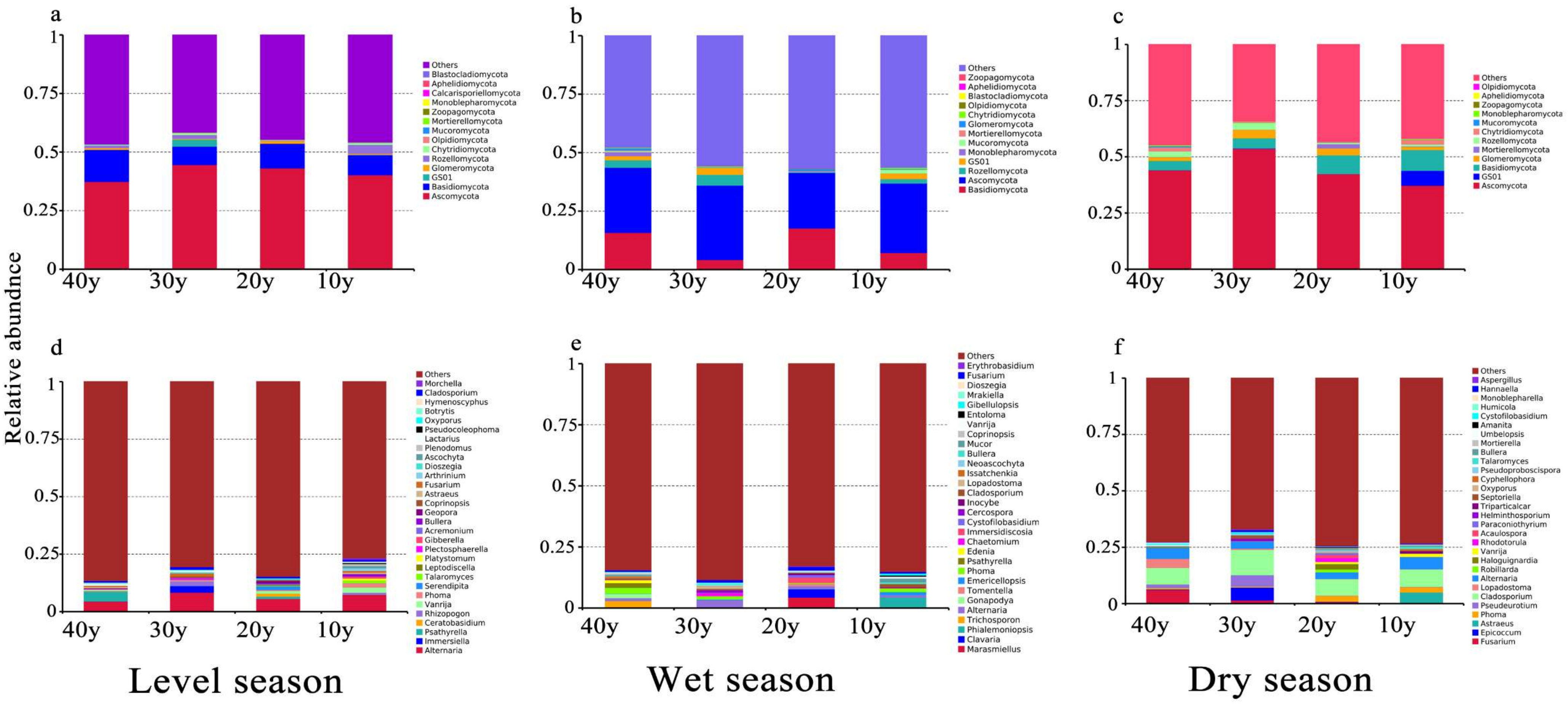

Figure S4. The relative abundance of fungal communities at the (a-c) phylum and (d-f) genus- levels in the reed rhizosphere in the level, wet and dry seasons.

References

Martin, M. (2011). Cutadapt removes adapter sequences from high-throughput sequencing reads. Embnet Journal, 17, 10-12. doi: org/10.14806/ej.17.1.200

Quast, C., Pruesse, E. Yilmaz, P., Gerken, J., Schweer, T., Yarza, P., Peplies, J., Glöckner, F.O. (2013). The SILVA ribosomal RNA gene database project: improved data processing and web-based tools. Nucleic. Acids Research 41, D590-D596. doi.org/10.1093/nar/gks1219

Edgar, R.C., Haas, B.J., Clemente, J.C., Quince, C., Knight, R. (2011). UCHIME improves sensitivity and speed of chimera detection. Bioinformatics, 27, 2194-2200. doi.org/10.1093/bioinformatics/btr381

Haas, B.J., Gever, D., Earl, A.M., Feldgarden, M., Ward, D.V., Giannoukos, G., Ciulla, D., Tabbaa, D., Highlander, S.K., Sodergren, E., Methe, B., Desantis, T.Z., the Human Microbiome Consortium, Petrosino, J.F., Knight, R., Birren, B.W. (2011). Chimeric 16S rRNA sequence formation and detection in Sanger and 454-pyrosequenced PCR amplicons. Genome research, 21, 494-504. doi: 10.1101/gr.112730.110.

Edgar, R.C. (2013). UPARSE: highly accurate OTU sequences from microbial amplicon reads. Nature methods, 10, 996-998. doi: org/10.1038/nmeth.2604

Edgar, R.C. (2004). MUSCLE: multiple sequence alignment with high accuracy and high throughput. Nucleic acids research, 32,1792-1797. doi.org/10.1093/nar/gkh340
